# Donanemab detects a minor fraction of amyloid-β plaques in post-mortem brain tissue of patients with Alzheimer’s disease and Down syndrome

**DOI:** 10.1007/s00401-022-02418-3

**Published:** 2022-04-16

**Authors:** Yvonne Bouter, Hendrik Liekefeld, Steffen Pichlo, Anna Celine Westhoff, Lydia Fenn, Preeti Bakrania, Thomas A. Bayer

**Affiliations:** 1 Department of Psychiatry and Psychotherapy, Division of Molecular Psychiatry, University Medical Center Göttingen (UMG), Georg-August-University, von-Siebold-Str. 5, 37075 Göttingen, Germany; 2 LifeArc, Centre for Therapeutics Discovery, Open Innovation Campus, Stevenage, UK

Donanemab, a humanized antibody against the N-truncated pyroglutamate amyloid-β peptide at position 3 (AβpE3), was recently assessed in a phase 2 trial for safety, tolerability and efficacy after passive immunization of patients with early Alzheimer’s disease (AD) [8]. The treatment demonstrated beneficial effects on the disease process with slowing cognitive and functional decline on all secondary clinical endpoints, reduced plaque load, and tau accumulation in a subgroup of patients analyzed by in vivo brain imaging*.* Early studies by Boche et al. [4] demonstrated a lower plaque load, and reduced tau aggregation in neuronal processes, but no evidence of beneficial effect on memory decline in a follow-up study of AD patients immunized with Aβ1–42. On the contrary, increased microgliosis and cerebral amyloid angiopathy (CAA) was observed. The link between plaque load and memory function is still a matter of controversial scientific debates, as plaque targeting treatment strategies did not convincingly improve cognition in a recent meta-analysis [1].

Antibodies against AβpE3 differ in their binding properties against soluble and aggregated conformations of AβpE3-42 [3]; therefore, it is important to understand whether they detect soluble oligomers, protofibrils and fibrillar amyloid within plaques and CAA as promising therapeutic targets. Once AβpE3-x monomers are generated, they adopt a pseudo β-hairpin structure at the N-terminus, which is specifically recognized by the TAPAS family of antibodies [2]. Pan-AβpE3 antibodies like 1–57 [15] react with a range of conformations: high-molecular weight oligomers, protofibrils and fibrillar forms found in different plaque types. Donanemab on the contrary has been claimed to react abundantly with amyloid plaques, especially with cored plaques [8].

Due to the lack of information on the binding of donanemab to pathological hallmarks in AD, we have performed an immunohistochemical study using post-mortem brain sections from patients with AD, Down syndrome and non-demented controls as well as AD mouse models 5XFAD, APP/PS1KI and TBA42. In temporal cortex brain sections of AD and Down syndrome cases, both pan-AβpE3 antibody 1–57 and donanemab detected only a fraction of plaques compared to the pan-Aβ antibody 2431–1 (Fig. 1, S1). In AD, the level for pan-AβpE3 was 63% and for donanemab only 37% of plaques positive for pan-Aβ. Interestingly, the level of donanemab versus pan-AβpE3 was significantly lower (*t* test *p* < 0.001). In Down syndrome, the situation was similar. The overall plaque load was higher than in AD cases and all plaques were positive for pan-Aβ. The pan-AβpE3 positive plaques accounted for 49%, and for donanemab, only 34% (lower donanemab-positive plaque load versus pan-AβpE3 did not reach statistical significance; *t* test, *p* = 0.09). The staining in control cases did not differ. Regarding vascular staining (CAA), donanemab and pan-AβpE3 staining appeared similar to pan-Aβ positive CAA (Fig. S1). Although donanemab reacted only with a fraction of amyloid plaques, it strongly detected the central core of plaques (Fig. S2). Semi-quantitative analysis of plaques (Figs. S3–S4) further supported the quantitative analysis (Fig. 1): donanemab showed the lowest binding capacity of amyloid plaques. While staining against pan-Aβ strongly reacted with all plaques, staining against pan-AβpE3 showed an intermediate pattern. Regarding semi-quantitative analysis of CAA, staining with the three antibodies did not show obvious differences between donanemab, pan-AβpE3 and pan-Aβ (Fig. S5). The demographics of human samples is shown in Table S1. In AD mouse models APP/PS1KI [7] and 5XFAD [10] (Figs. S6, S7), donanemab detected plaques. However, the immunoreactivity was significantly lower as compared to pan-Aβ in the 5XFAD model (Fig. S7). Interestingly, donanemab also detected intraneuronal AβpE3-42 in TBA42 [16] and APP/PS1KI mouse brains (Fig. S6). Using ELISA antibody binding assays, we demonstrated that donanemab reacts with AβpE3-42, but not with Aβ1–42 and Aβ4–42 (Fig. S8). The present study might be limited in the use of AD mouse models. A major scientific advancement is the development of APP knock-in mouse models by the group of Takaomi Saido. These knock-in mice express the Swedish and Beyreuther/Iberian mutations with and without the Arctic mutation in the APP gene [13]. Due to the use of the endogenous mouse APP promoter, the expression is cell-type and temporal specific. The APP^NL−F^ model, for example, expresses APP at wild-type levels while producing pronounced elevation of Aβ42 due to the combined effect on APP proteolysis of the Swedish and Iberian mutations.

**Fig. 1:**
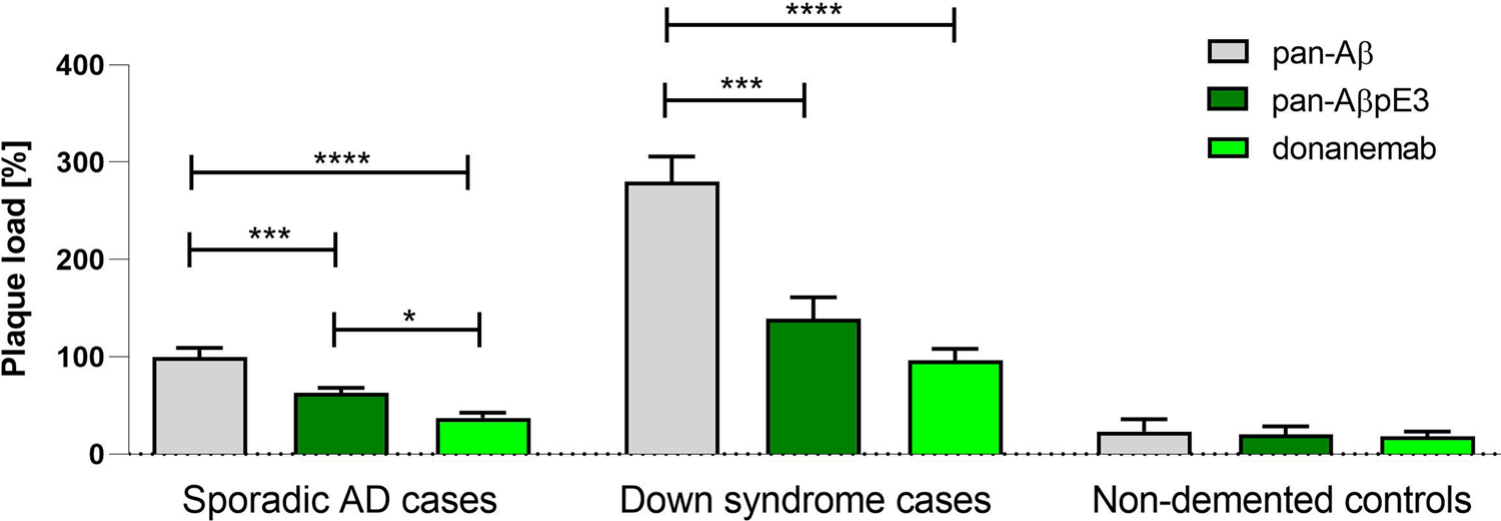
Plaque load quantification in temporal cortex of cases with AD, Down syndrome and non-demented controls. Plaque load staining with pan-Aβ antibody 2431–1 in AD was used as reference. Both pyroglutamate Aβantibodies 1–57 (pan-AβpE3) and donanemab significantly detected less plaques. Of note, donanemab showed the lowest plaque load. In Down syndrome cases, again both pan-AβpE3 and donanemab detected only a fraction of plaques compared to pan-Aβ staining. The difference between pan-AβpE3 and donanemab staining did not reach statistical significance. No difference between the three antibodies was observed in non-demented controls. One-way analysis of variance (ANOVA) followed by Bonferroni multiple comparisons (F = 22.91; *p* < 0.0001; R squared = 0.3430). *p < 0.05; ***p < 0.001, ****p < 0.0001; data presented as mean ± SEM

The risk of amyloid-related imaging abnormalities with edema and effusions (ARIA-E) [14] is a major concern of treating AD patients with antibodies recognizing plaques. ARIA-E has been reported in a small group of AD patients treated with donanemab, which could be a consequence of the lower plaque binding activity [8]. Recently, it has been shown that targeting the pseudo β-hairpin structure of AβpE3 monomers by neutralizing antibodies is sufficient to reduce plaque load and rescue glucose metabolism, memory deficits as well as neuron loss in AD mouse models, although this epitope is not found in plaques [2]. The antibodies against AβpE3 differ in their binding properties against soluble and aggregated conformations of AβpE3-42 [3]. Therefore, it is of upmost therapeutic importance whether antibodies detect soluble oligomers, protofibrils and/or fibrillar amyloid within plaques and CAA. Saido et al*.* [12] demonstrated for the first time that AβpE3 production and retention is an early and critical event in senile plaque formation in AD and Down syndrome patients. Subsequent studies confirmed the importance of AβpE3 in AD [5, 6, 11]. Importantly, the deposition of AβpE3 was reported to be directly linked with hyperphosphorylated tau and neuropathological staging of AD [7] as well as in related mouse models [9], clearly supporting its role as a potential drug target against AD.

## Supplementary Information

Below is the link to the electronic supplementary material.

Supplementary file1 (DOCX 2203 KB)
