# Supplementary material for: Donanemab detects a minor fraction of amyloid-β plaques in post-mortem brain tissue of patients with Alzheimer’s disease and Down syndrome
Source: Acta Neuropathol. 2022 Apr 16;143(5):601–3. doi: 10.1007/s00401-022-02418-3 (PMC9038931; doi:10.1007/s00401-022-02418-3)
Supplement: Supplementary file 1 — Supplementary file1 (DOCX 2203 KB) [file 401_2022_2418_MOESM1_ESM.docx]

**Supplemental material**

**Figure S1 Immunohistochemical staining pattern of sporadic AD brains.** Abundant plaque pathology could be recognized in the cortex with the pan-Aβ antibody 2431-1 (**a**, **d**), pan-AβpE3 antibody 1-57 (**b**, **e**), and donanemab (**c**, **f**) with donanemab recognizing only a fraction of plaques. Cerebral amyloid angiopathy (CAA) was visualized with 2431-1 (**g**), 1-57 (**h**) and donanemab (**f**) demonstrating comparable CAA staining pattern in cortical parallel sections. Scale bar: 100µm.


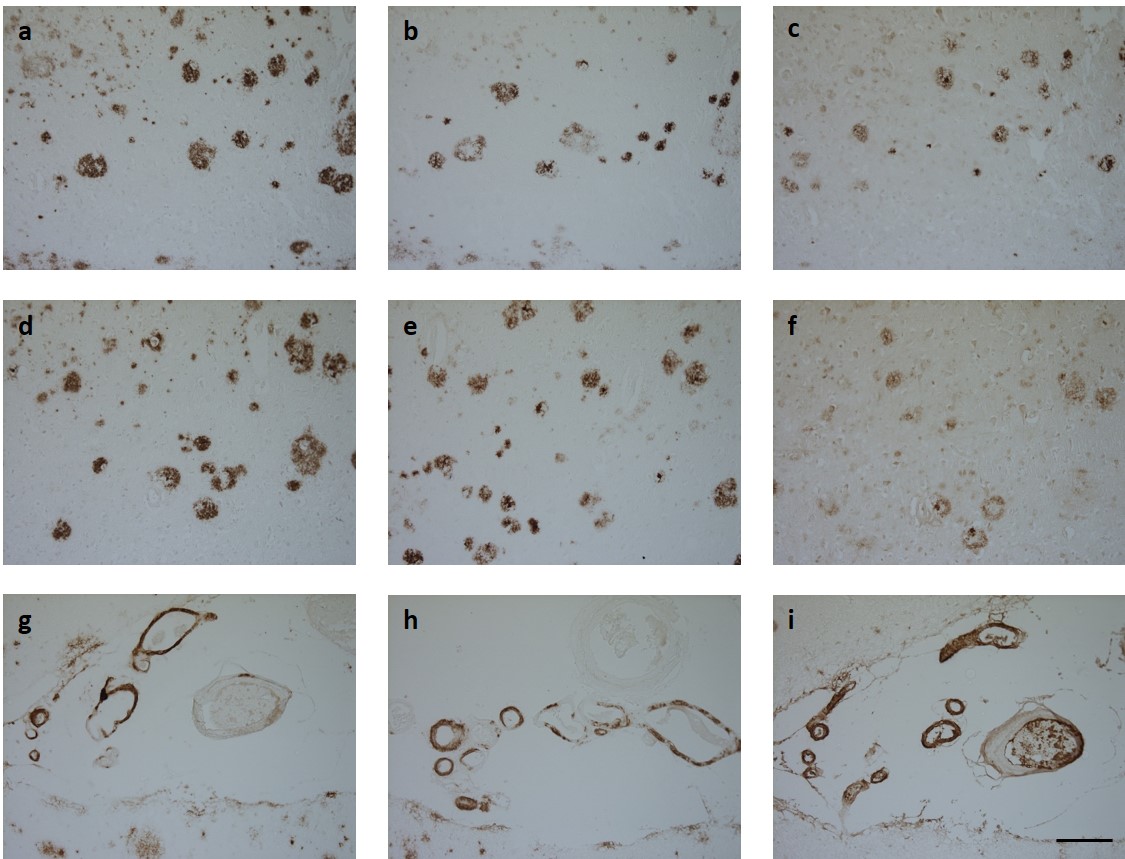


**Figure S2** **Double-Staining with Thioflavin-S with pan-Aβ antibody 2431-1 or donanemab.** Plaque pathology in the temporal cortex of a AD case visualized with thioflavin-S (**a**, **b**) and 2431-1 (**c**) or donanemab (**d**). (**e**) Overlay of **a** and **c**. (**f**) Overlay of **b** and **d**. Thioflavin-S was used as a co-stain for fibrillar Aβ after antibody staining. Scale bar: 50µm.


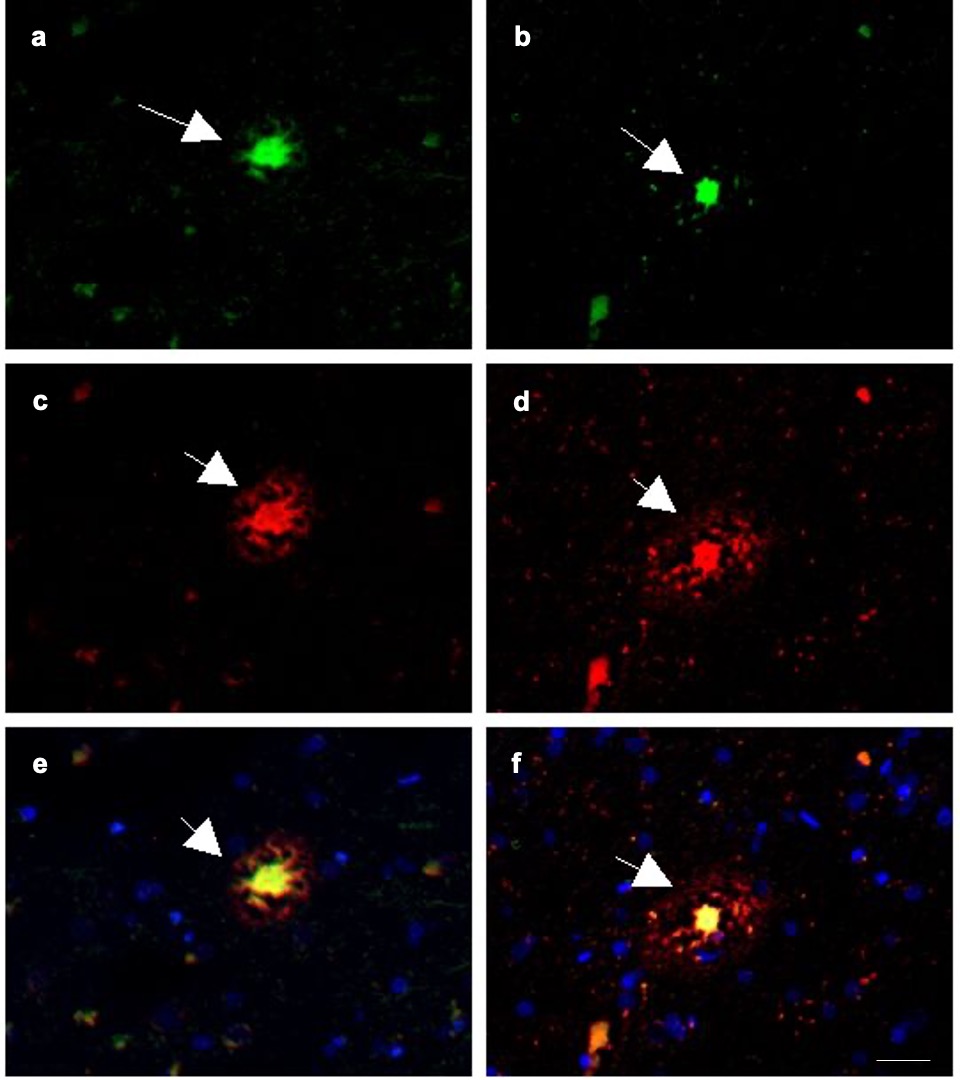


**Figure S3 Example pictures for semi-quantitative assessment using pan-Aβ antibody 2431-1.** Aβ staining intensity: no staining (-; no plaques per mm^2^); barely perceptible staining ((+); < 5 plaques per mm^2^); weak staining (+; < 20 plaques per mm^2^); moderate staining (++; < 40 plaques per mm^2^); intense staining (+*+; > 40 plaques per mm^2^). Scale bar: 100 µm.


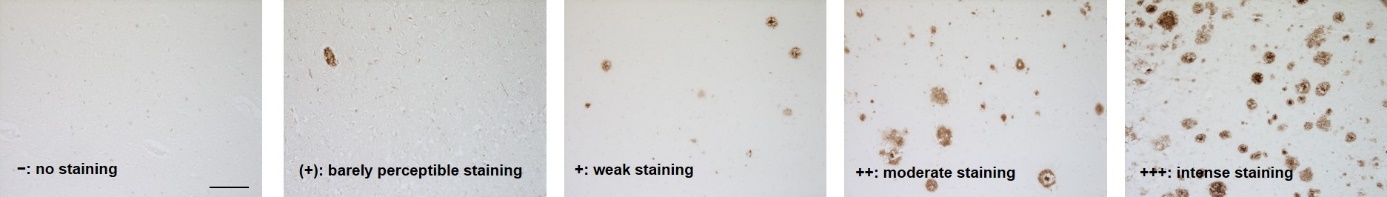


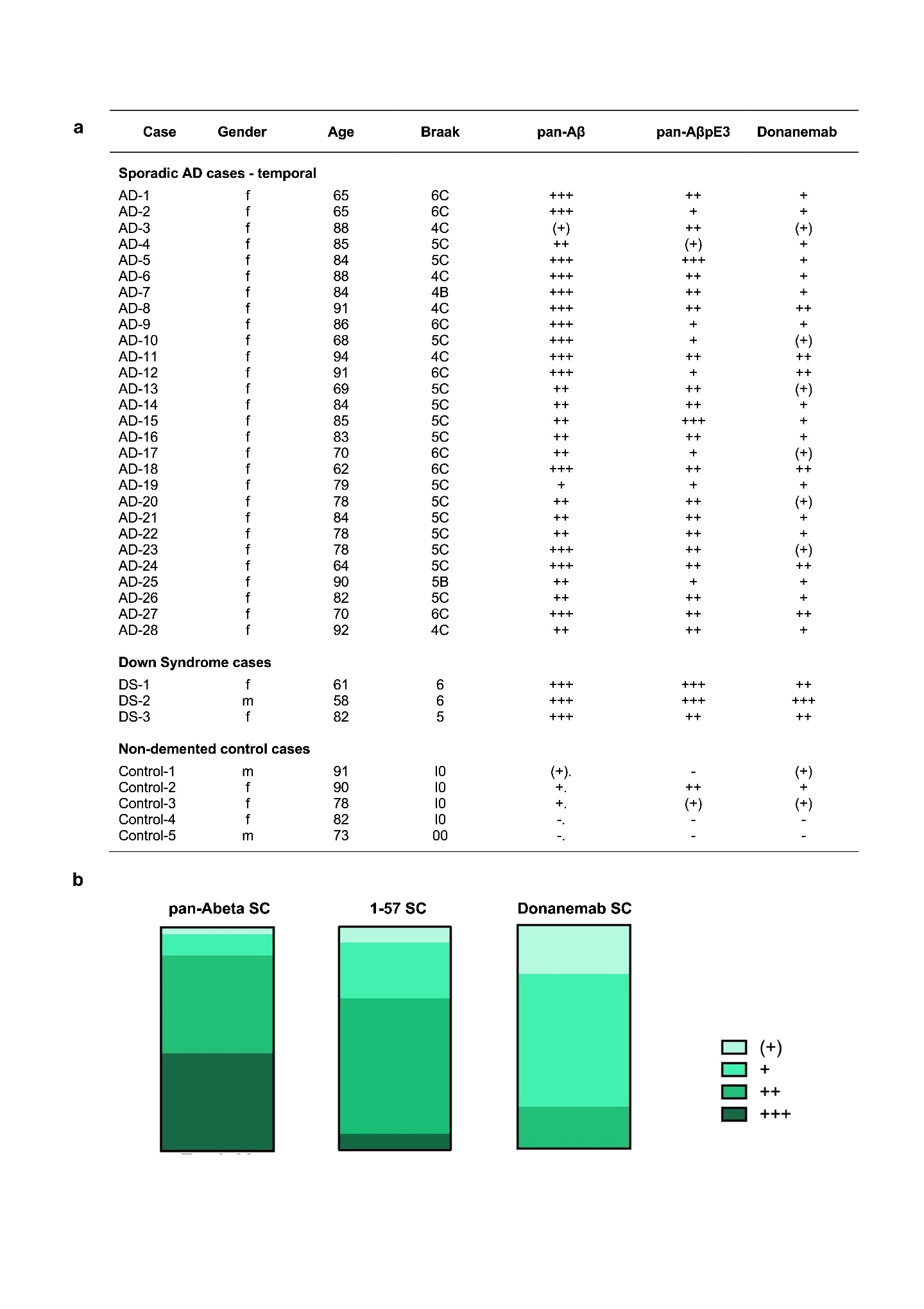
**Figure S4** **Semi-quantitative assessment of plaque pathology in the temporal cortex of sporadic cases of AD, Down syndrome and non-demented control cases.** The semi-quantitative plaque load assessment provides similar results as the quantitative plaque load assessment presented in figure 1 with significantly lower staining with donanemab versus 2431-1. (**a**) List of demographic data and staining profiles of donanemab, pan-AβpE3 antibody 1-57 and the pan-Aβ antibody 2431-1 in sporadic AD patients, Down syndrome and non-demented control cases. (**b**) Summary of plaque staining profiles of 2431-1 and donanemab. Aβ staining intensity: (+) barely perceptible staining; + weak staining; ++ moderate staining; +++ intense staining. Abbreviations: sporadic cases (SC) of AD; Down syndrome (DS).


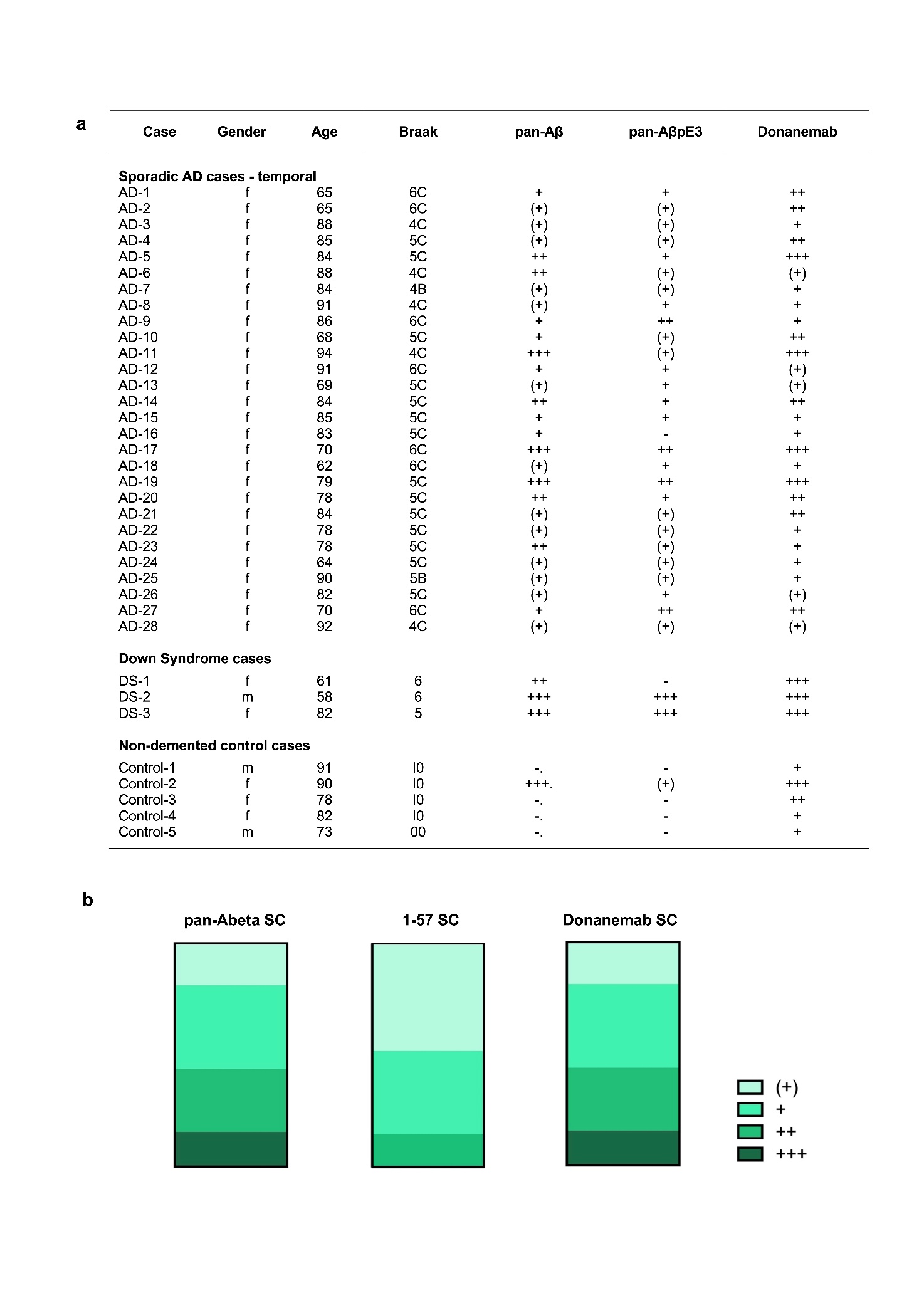
**Figure S5 Semi-quantitative assessment of congophilic amyloid angiopathy (CAA) in temporal cortex of sporadic cases of AD, Down syndrome and non-demented control cases.** The semi-quantitative CAA assessment demonstrates that CAA staining with donanemab and 2431-1 are similar. (**a**) List of demographic data and staining profiles of donanemab, pan-AβpE3 antibody 1-57 and the pan-Aβ antibody 2431-1 in sporadic AD patients, Down syndrome and non-demented control cases. (**b**) Summary of CAA staining profiles of 2431-1 and donanemab. Aβ staining intensity: (+) barely perceptible staining; + weak staining; ++ moderate staining; +++ intense staining. Abbreviations: sporadic cases (SC) of AD; Down syndrome (DS).

**Figure S6** **Immunohistochemical staining of APP/PS1KI and TBA42 mouse brain.** Immunostaining with 2431-1 (**a**) and donanemab (**b**) demonstrating intraneuronal Aβ accumulation and extracellular plaques in the hippocampus of 2-month-old APP/PS1KI mice. Positive intraneuronal immunoreactivity could be detected with 2431-1 (**c**) and donanemab (**d**) in the CA1 region of the hippocampus of 3-month-old TBA42 mice. Staining in both models is less abundant with donanemab. Scale bar: 200µm


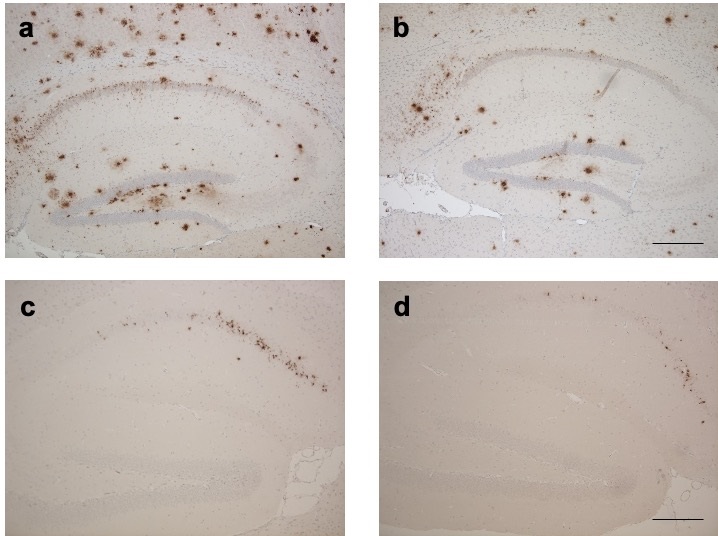


**Figure S7 Immunohistochemical staining of 5XFAD mouse brain.** Donanemab and pan-AβpE3 antibody 1-57 detected significantly less plaques in (**a**) hippocampus and (**b**) cortex of 5-month-old 5XFAD than the pan-Aβ antibody 24311. Antibody 2431-1 showed abundant plaque pathology in the hippocampus (**c**) and cortex (**f**) of 5XFAD mice. Plaque staining was less abundant with pan-AβpE3 antibody in (**d**) hippocampus and (**g**) cortex, as well as with donanemab in hippocampus (**e**) and (**h**) cortex. Donanemab was predominantly in the core of plaques (**e**, **h**). One-way analysis of variance (ANOVA) followed by Bonferroni multiple comparisons (F=323.1 and 195.7; p<0.0001; R squared=0.7521 and 0.8501). **p < 0.01, ***p < 0.001; data presented as mean ± SEM. (**h**) Scale bar: (**c-e**) 200 µm; (**f-h**)100 µm.


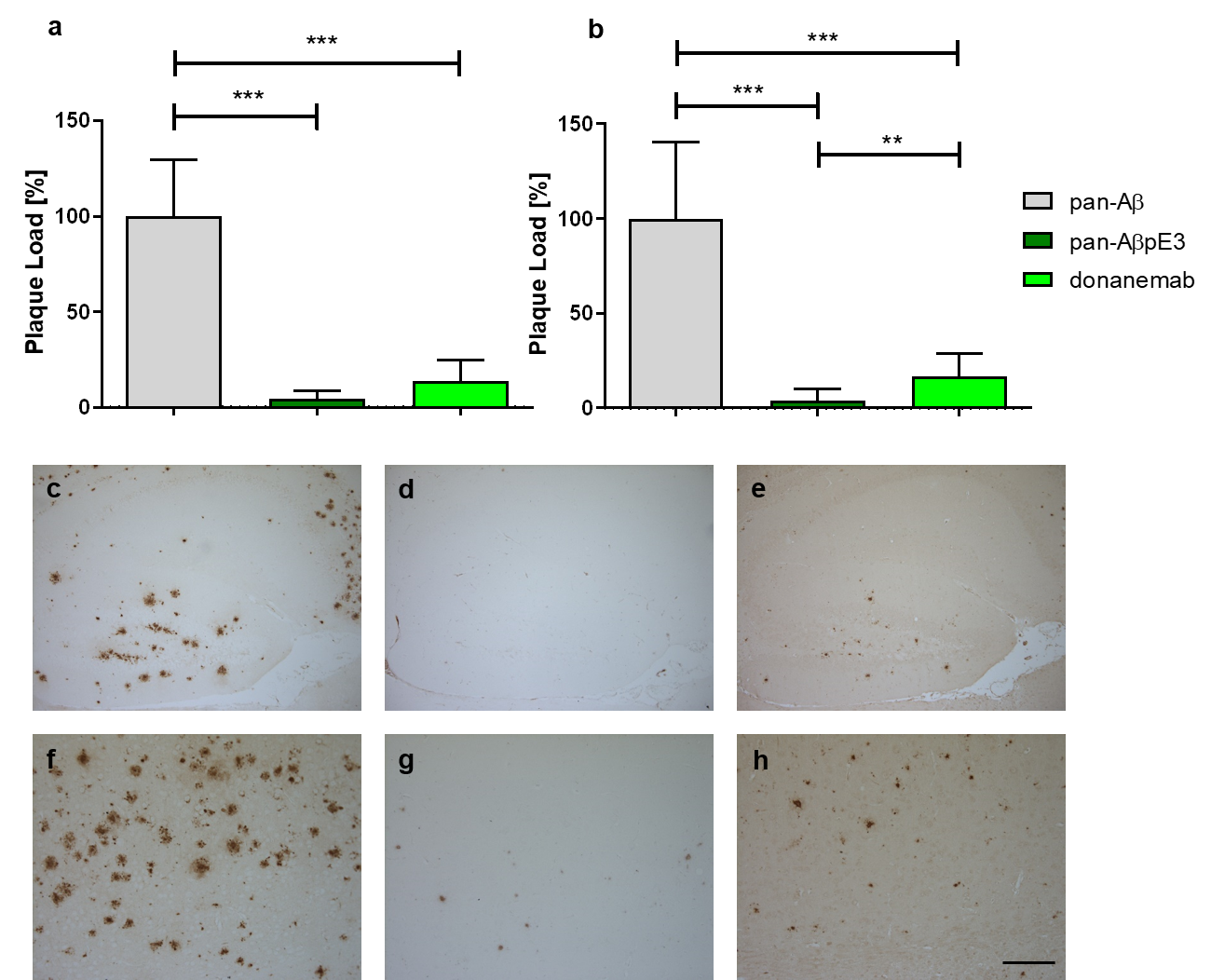


**Figure S8** **The binding of donanemab characterized by ELISA assays using Aβ1-42, AβpE3-42 and Aβ4-42 peptides as antigens.** Donanemab was found to show high affinity binding to AβpE3-42 (HuG1K EC50 = 0.018 nM) and weakly to Aβ4-42 at high concentrations (EC50 not determined within these concentration range limits as >2uM). No interaction was seen with Aβ1-42, which clearly confirms the unique specificity of donanemab. Donanemab was cloned into a human IgG1 background vector. The data shown were fitted to a non-linear regression four parameter model using Prism.

**
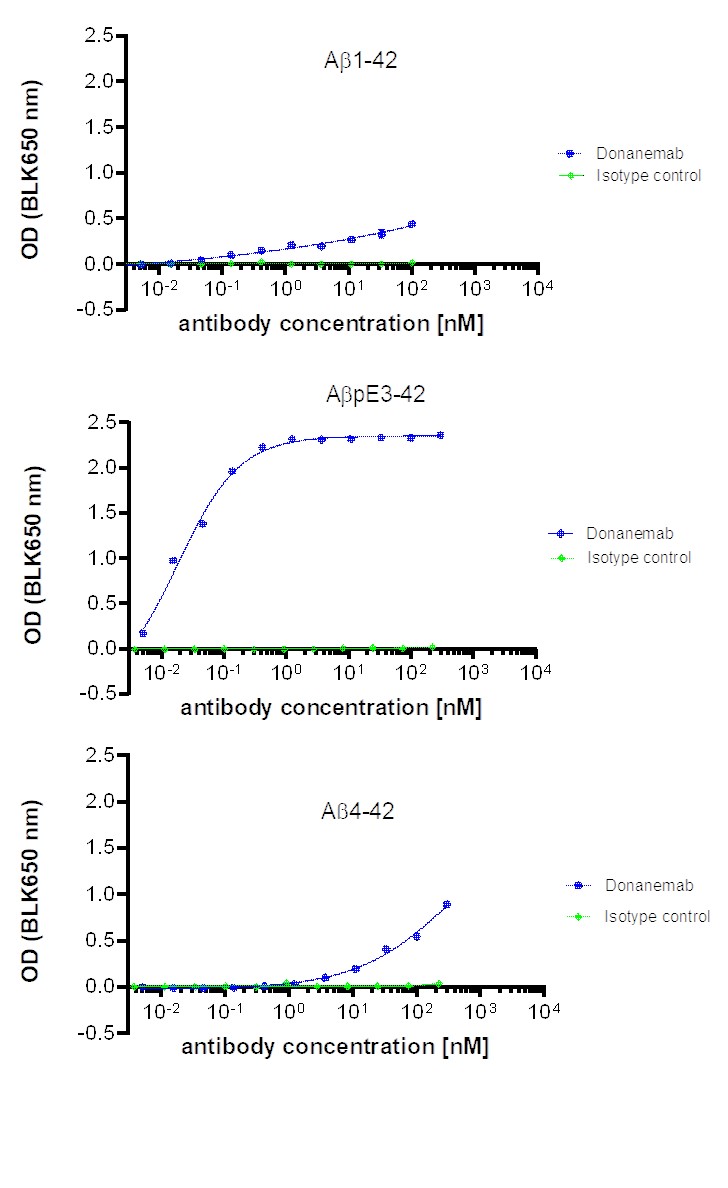
**

**Table S1** **Demographical data of human samples.** Abbreviations: AD, Alzheimer’s disease case; DS, Down syndrome case; f, female; m, male.

| **Case** | **Gender** | **Age of first symtoms** | **Age of death** | **Reisberg scale** | | **Braak** | **ApoE** | | |  |
| --- | --- | --- | --- | --- | --- | --- | --- | --- | --- | --- |
| **Sporadic AD cases** | | | |  | |  |  | | |  |
| AD-1 | f | 49 | 65 | 7 | | 6C | 33 | |  |  |
| AD-2 | f | 55 | 65 | 7 | | 6C | 33 | |  |  |
| AD-3 | f | 84 | 88 | 7 | | 4C | 33 | |  |  |
| AD-4 | f | 53 | 62 | 7 | | 6C | 43 | |  |  |
| AD-5 | f | 49 | 58 | 7 | | 6C | 43 | |  |  |
| AD-6 | f | 76 | 88 | 7 | | 4C | 33 | |  |  |
| AD-7 | f | 80 | 84 | 7 | | 4B | 32 | |  |  |
| AD-8 | f | 81 | 86 | 6 | | 6C | 44 | |  |  |
| AD-9 | f | 86 | 94 | 6 | | 4C | 32 | |  |  |
| AD-10 | f | 78 | 84 | 6 | | 5C | 33 | |  |  |
| AD-11 | f | 74 | 78 | 7 | | 5C | 33 | |  |  |
| AD-12 | f | 89 | 92 | 4 | | 4C | 32 | |  |  |
| AD-13 | f | 84 | 91 | 5 | | 4C | 43 | |  |  |
| AD-14 | f | n.a. | 68 | n.a. | | 5C | 33 | |  |  |
| AD-15 | f | 86 | 91 | 7 | | 6C | 33 | |  |  |
| AD-16 | f | 65 | 69 | 6 | | 5C | 42 | |  |  |
| AD-17 | f | 67 | 84 | n.a. | | 5C | 43 | |  |  |
| AD-18 | f | 70 | 85 | 7 | | 5C | 44 | |  |  |
| AD-19 | f | 53 | 70 | 7 | | 6C | 33 | |  |  |
| AD-20 | f | 73 | 79 | 7 | | 5C | 33 | |  |  |
| AD-21 | f | 80 | 88 | 7 | | 5C | 33 | |  |  |
| AD-22 | f | 68 | 78 | 5 | | 5C | 44 | |  |  |
| AD-23 | f | n.a. | 64 | 7 | | 5C | 42 | |  |  |
| AD-24 | f | 76 | 86 | 6 | | 4C | 43 | |  |  |
| AD-25 | f | 61 | 70 | 6 | | 6C | 44 | |  |  |
| AD-26 | f | 78 | 85 | 7 | | 5C | 43 | |  |  |
| AD-27 | f | 81 | 84 | 7 | | 5C | 33 | |  |  |
| AD-28 | f | 81 | 88 | 6 | | 4C | n.a. | |  |  |
|  |  |  |  |  | |  |  | |  |  |
| **Down syndrome cases** | | | | | |  |  | |  |  |
| DS-1 | f | 53 | 61 | 7 | | 6 | n.a. | |  |  |
| DS-2 | m | 49 | 58 | 7 | | 6 | n.a. | |  |  |
| DS-3 | f | 72 | 82 | 6 | | 5 | n.a. | |  |  |
|  |  |  |  |  | |  |  | |  |  |
| **Non-demented control cases** | | |  |  | |  |  | |  |  |
| Control-1 | m | - | 91 | n.a. | | I0 | n.a. | |  |  |
| Control-2 | f | - | 90 | n.a. | | I0 | n.a. | |  |  |
| Control-3 | f | - | 78 | n.a. | | I0 | n.a. | |  |  |
| Control-4 | f | - | 82 | n.a. | | I0 | n.a. | |  |  |
| Control-5 | m | - | 73 | n.a. | | 00 | n.a. | |  |  |
|  |  |  |  | |  | |  |  |  |  |
|  |  |  |  |  | |  |  | | |  |
|  |  |  |  |  | |  |  | | |  |
|  |  |  |  |  | |  |  | | |  |
|  |  |  |  |  | |  |  | | |  |
|  |  |  |  |  | |  |  | | |  |

**Material and Methods**

**Human samples**

Brain sections of the temporal cortex of 28 patients with sporadic AD (females, mean age ± SEM 79.43 ± 1.9; Braak stage 4–6; ApoE4: 12/28), five non-demented control subjects (3 female/2 males, mean age ± SEM 82.8 ± 3.4; Braak stage 0–1) and three Down syndrome cases (females, mean age ± SEM 67 ± 7.5; Braak stage 5–6) were examined (Table S1). All human brain samples were obtained from the Netherlands Brain Bank (NBB, Amsterdam, The Netherlands). NBB works with a rapid autopsy program to minimize postmortem delay (PMD). Definite diagnosis was based on established criteria and written informed consent was obtained from all subjects. The control subjects did not suffer from any primary neurological or psychiatric disease or brain metastases nor did they have a history of medication or drug treatment. The inclusion as a control donor is based both on review of medical records and on the Braak stage by the Netherlands Brain Bank. All sporadic AD patients had Reisberg Scales of four or higher. The Reisberg Scale is used by caregivers as clinical diagnostic criteria and divided into seven different stages. Stages 4–7 are dementia stages, while stages 1–3 are pre-dementia stages [3].

**Animal models**

In this study three established AD-like transgenic mouse lines were used: TBA42 [7], 5XFAD [5] and APP/PS1KI [3]. The 5XFAD double transgenic mouse model (Jackson Laboratories, Bar Harbor, ME, USA) carries five mutations found in patients with familial AD. 5XFAD mice overexpress the 695 amino acid isoform of the human amyloid precursor protein (APP695) carrying the Swedish, London and Florida mutations under the control of the murine Thy1-promoter. In addition, human presenilin-1 (PSEN-1) carrying the M146L/L286V mutations is expressed under the control of the murine Thy1- promoter [5]. 5XFAD mice used in this study were kept on a C57Bl/6J genetic background. The APP/PS1KI transgenic mouse model (a generous gift by Dr. Laurent Pradier, Sanofi, Paris) carries M233T/L235P knocked-in mutations in presenilin-1 and overexpresses mutated human amyloid precursor protein carrying the London and Swedish mutations under the Thy-1 promoter [3]. APP/PS1KI mice used in this study were kept on a C57Bl/6J genetic background. TBA42 mice express Aβ3Q−42 fused to the murine TRH signal peptide under the control of the neuronal Thy-1 promoter. The glutamate at position three of the Aβ amino acid sequence is mutated into glutamine to facilitate enhanced pyroglutamate formation. TBA42 mice express unmodified Aβ3Q−42, which can be readily converted to AβpE3−42 by glutaminyl cyclase [4, 7].

**ELISA assays of donanemab binding to Aβ peptides**

ELISA antibody binding assays were carried out using standard protocols with a selection of Aβ peptides immobilized on plates for Aβ1-42, AβpE3-42 and Aβ4-42. Briefly, Aβ peptides were coated on to 384 well plates, blocked, incubated with Donanemab for 1hr at 37^o^C, secondary-HRP conjugated antibodies were added and incubated for 1hr at 37^o^C. Plates were developed using K-BLUE substrate (20ul/well) and RED-STOP (10ul/well) solution. Optical density at 650 nm was measured using the Pherastar Plus as described [1].

**Immunohistochemistry and antibodies**

Brains were embedded in paraffin and cut into sections of 4 μm. Immunohistochemistry was carried out as previously described [2]. The pan-Aβ antibody 2431-1 (1:500), pan-AβpE3 antibody 1-57 (1:5000, 1.5 mg/ml) [6] and the biosimilar antibody donanemab (cloned into human IgG1, 1:2000; 1.5 mg/ml) were used. The primary sequence of the biosimilar donanemab is identical to the original donanemab antibody. However, changes in the manufacturing process ranging from a change in the supplier of cell culture media to new purification methods or new manufacturing sites can be different. Corresponding biotinylated anti-rabbit and anti-human antibodies (DAKO (Glostrup, Denmark) were used as secondary antibodies. Staining was visualized using the ABC method with a Vectastain Kit (Vector Laboratories, Burlingame, CA, USA) and diaminobenzidine as chromogen. Images were taken with an Olympus BX51 microscope equipped with a MoticamPro 282B digital camera. The semiquantitative analysis of plaque pathology and congophilic amyloid angiopathy (CAA) in paraffin-embedded tissue was based on Aβ staining intensity according to the following scale: −: no staining; (+): barely perceptible staining; +: weak staining; ++: moderate staining; +++: intense staining. All slides were assessed by three observers with no significant inter-observer variability.

Double-immunofluorescence staining was visualized using Alexa Fluor 488- and Alexa Fluor 594-conjugated secondary antibodies (Molecular Probes, Eugene, OR, USA) and 4′,6-diamidino-2-phenylindoleThioflavin-S was used to co-stain for fibrillar Aβ after antibody staining.

**Amyloid plaque load quantification**

Plaque load was quantified in human brain samples diagnosed with sporadic AD and 5XFAD mice. For each of the human cortical brain sections four paraffin-embedded sections were stained simultaneously with DAB as chromogen. The relative Aβ load was evaluated using an Olympus BX-51 microscope equipped with an Olympus DP-50 camera and the ImageJ software (NIH, USA). Representative pictures of 20x magnification were systematically captured. In 5XFAD mice, plaque load in the cortex was evaluated capturing serial images of 20x magnification on three sections per animal which were 30 μm afar from each other. Using ImageJ (V 1.51, NIH, Bethesda, MA, USA) the pictures were binarized to 8-bit black and white images and a fixed intensity threshold was applied defining the DAB staining. The percentage area covered by positive DAB staining was measured for each image.

**Statistical analysis**

The statistical significance of differences between groups were tested with one-way analysis of variance (ANOVA) followed by Bonferroni multiple comparisons and by t-test. All data were reported as the mean with either standard error of the mean (SEM). All statistics were calculated using GraphPad Prism version 9 for Mac (GraphPad Software, San Diego, CA, USA).

**References**

1 Bakrania P, Hall G, Bouter Y, Bouter C, Beindorff N, Cowan R et al (2021) Discovery of a novel pseudo β-hairpin structure of N-truncated amyloid-β for use as a vaccine against Alzheimer’s disease. Mol Psych: <https://doi.org/10.1038/s41380-021-01385-7>

2 Bouter Y, Dietrich K, Wittnam JL, Rezaei-Ghaleh N, Pillot T, Papot-Couturier S et al (2013) N-truncated amyloid beta (Abeta) 4-42 forms stable aggregates and induces acute and long-lasting behavioral deficits. Acta Neuropathol 126: 189-205. <https://doi.org/10.1007/s00401-013-1129-2>

3 Casas C, Sergeant N, Itier JM, Blanchard V, Wirths O, van der Kolk N et al (2004) Massive CA1/2 neuronal loss with intraneuronal and N-terminal truncated Abeta 42 accumulation in a novel Alzheimer transgenic model. Am J Pathol 165: 1289-1300. <https://doi.org/10.1016/s0002-9440(10)63388-3>

4 Meissner JN, Bouter Y, Bayer TA (2015) Neuron Loss and Behavioral Deficits in the TBA42 Mouse Model Expressing N-Truncated Pyroglutamate Amyloid-beta3-42. J Alz Dis 45: 471-482. <https://doi.org/10.3233/JAD-142868>

5 Oakley H, Cole SL, Logan S, Maus E, Shao P, Craft J et al (2006) Intraneuronal beta-Amyloid Aggregates, Neurodegeneration, and Neuron Loss in Transgenic Mice with Five Familial Alzheimer's Disease Mutations: Potential Factors in Amyloid Plaque Formation. J Neurosci 26: 10129-10140. <https://doi.org/10.1523/JNEUROSCI.1202-06.2006>

6 Wirths O, Bethge T, Marcello A, Harmeier A, Jawhar S, Lucassen PJ et al (2010) Pyroglutamate Abeta pathology in APP/PS1KI mice, sporadic and familial Alzheimer's disease cases. J Neural Transm (Vienna) 117: 85-96. https://doi.org/10.1007/s00702-009-0314-x

7 Wittnam JL, Portelius E, Zetterberg H, Gustavsson MK, Schilling S, Koch B et al (2012) Pyroglutamate amyloid β (Aβ) aggravates behavioral deficits in transgenic amyloid mouse model for Alzheimer disease. J Biol Chem 287: 8154-8162. <https://doi.org/10.1074/jbc.M111.308601>
